# Using Gas-Driven Shock Tubes to Produce Blast Wave Signatures

**DOI:** 10.3389/fneur.2020.00090

**Published:** 2020-02-21

**Authors:** Rubbel Kumar, Ashish Nedungadi

**Affiliations:** ^1^The Johns Hopkins University Applied Physics Laboratory, Laurel, MD, United States; ^2^Department of Aerospace Engineering, University of Maryland, College Park, MD, United States

**Keywords:** primary blast injury, shock tube, blast wave, CFD, shock tube parameters

## Abstract

The increased incidence of improvised explosives in military conflicts has brought about an increase in the number of traumatic brain injuries (TBIs) observed. Although physical injuries are caused by shrapnel and the immediate blast, encountering the blast wave associated with improvised explosive devices (IEDs) may be the cause of traumatic brain injuries experienced by warfighters. Assessment of the effectiveness of personal protective equipment (PPE) to mitigate TBI requires understanding the interaction between blast waves and human bodies and the ability to replicate the pressure signatures caused by blast waves. Prior research has validated compression-driven shock tube designs as a laboratory method of generating representative pressure signatures, or Friedlander-shaped blast profiles; however, shock tubes can vary depending on their design parameters and not all shock tube designs generate acceptable pressure signatures. This paper presents a comprehensive numerical study of the effects of driver gas, driver (breech) length, and membrane burst pressure of a constant-area shock tube. Discrete locations in the shock tube were probed, and the blast wave evolution in time at these points was analyzed to determine the effect of location on the pressure signature. The results of these simulations are used as a basis for suggesting guidelines for obtaining desired blast profiles.

## Introduction

Detonation of explosive devices is typically associated with shrapnel and fire, both of which lead to visible injuries. Improvised explosive devices (IEDs) may primarily produce blast waves and shocks, which can lead to injuries that are superficially undetectable. The increased incidence of IEDs in military conflicts has been associated with a major increase in the number of traumatic brain injuries (TBIs) (1). The increased prevalence of this injury has led to a heightened need to investigate both the mechanisms by which the injury occurs and the need for personal protective equipment (PPE) (e.g., helmet systems). To conduct these analyses effectively, it is important to replicate the blast overpressures experienced by warfighters in theater, through either blast testing or laboratory-based methods. Although blast events through explosive detonation may better replicate real-world scenarios, these methods are costly, inherently limited in repeatability, and therefore not conducive to a large number of tests (2). Laboratory test methods using shock tubes offer more controlled, repeatable, and less expensive platforms for assessing blast traumatic brain injuries (bTBI) and performance of PPE. Furthermore, shock tubes can be configured to generate blast signatures representative of free-field events (3).

Although compression-driven shock tubes have been validated as a method of generating representative blast waves (3), blast waves produced by shock tubes depend on a number of design parameters, including the driver gas, the driver (breech) length, and the membrane burst pressure (pressure at which the membrane separating the pressurized driver section from the driven section bursts) (4). The Friedlander shock profile is a good target waveform to replicate through laboratory-based methods because it is a theoretical approximation and neglects the effects of reverb, secondary shocks, ground balance, and reflections. Additional complexities exist in field-generated blast test data that may be difficult to replicate experimentally. The Friedlander blast wave profile is described by the modified Friedlander equation,

$$\begin{array}{l} P=P_{0}\left(1-\frac{t}{t_{d}}\right)e^{\frac{-bt}{t_{d}}} \end{array}$$

where P_0_ is the peak pressure, t_d_ is the duration of the positive phase, and b is the exponential constant that controls the rate of decay (5).

**Figure 1:**
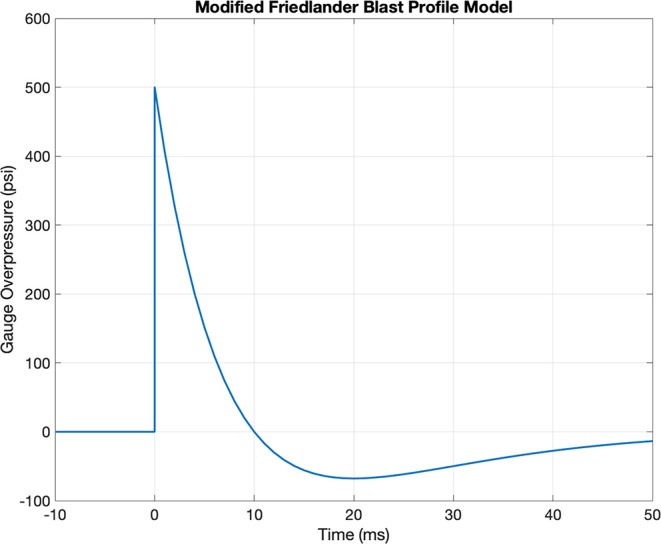
Example Friedlander wave profile with b = 1, P_0_ = 500 psi, and t_d_ = 10 ms.

Figure 1 shows a profile obtained with a typical set of input parameters. Thus, a person subjected to a blast wave first experiences a sharp pressure rise due to the passage of the shock front, followed by an exponential decay, and finally a rarefaction wave (6). The positive phase of the wave is the period between the initial pressure rise to the time the pressure returns to ambient conditions. Similarly, the negative phase of the wave is the period between when the pressure drops below the ambient conditions to the time the pressure once again returns to ambient conditions.

There has been considerable research on the use of shock tubes to further the understanding of how injuries are caused by blast waves. Much of this work began with efforts to improve fundamental knowledge of how shocks propagate within shock tubes as a function of design parameters. For example, Reneer et al. conducted experiments using a multi-mode shock tube to study how the use of different driver gases affects the pressure signature and what injuries these caused to a rat brain in the test section (7). Sundaramurthy and Chandra also studied the impact of different driver gases on the shock profile, in addition to conducting parametric studies that varied the shock tube breech length and burst pressure (4). They concluded that a compressed-gas shock tube can be used to simulate primary blast injury for blast-induced neurotrauma studies.

Other researchers studied how flow properties differ inside and outside a shock tube to assess the validity of testing outside a shock tube, called “end-jet testing.” Chandra et al. studied the shock evolution within and outside shock tubes, concluding that the placement of a test article outside of the open end of a shock tube exposed the test article to complex flow phenomena that were not representative of blast waves in the field (3). Kuriakose et al., Yu et al., and Needham et al., also studied the effects of the test article's placement and whether end-jet testing provided representative results (8–10). Kuriakose et al. (8) and Needham et al. (10) agreed with Chandra's conclusions, while Yu et al. (9) concluded that end-jet testing was acceptable under specific constraints. Specifically, Yu et al. concluded that testing inside is acceptable as long as the test article is placed at least 8–10 shock tube diameters down the driven section, measured from the diaphragm, and testing outside is acceptable as long as the test article is placed within ½ a tube diameter from the exit of the shock tube (3, 8, 9). When testing inside, another important consideration is the blockage, “the ratio of the total ‘presented area' of the obstruction relative to the cross-section of the tube,” and Needham et al. found that the blockage should not exceed 10–25% depending on the exact nature of the experiment (10).

For a number of years, personnel at the Johns Hopkins University Applied Physics Laboratory (JHU/APL) have conducted studies to improve their ability to replicate blast wave scenarios that cause injuries (11). They used both shock tubes and free-field testing to characterize shocks generated by IEDs. This work has typically been limited to performance assessment of existing laboratory shock tube designs.

The objective of the work described in this paper is to use computational fluid dynamics (CFD) simulations to characterize the primary overpressure blast environment and how it changes based on the design of a laboratory shock tube. Studies were conducted to understand the effects of varying the membrane driver length, burst pressure, and driver gas. These studies focused on how the pressure-time history varies within a shock tube and do not include how the environment changes the wave after the shock tube's exit. Conducting CFD simulations is less expensive, faster, and therefore better suited for the parametric studies than building an array of modular shock tubes to conduct laboratory experiments. The results from these analyses can be used by experimentalists to ensure that the shock tube design and test article placement are conducive to producing the desired blast exposure.

## Materials and Methods

The shock tube in this study consists of a high-pressure region (the driver section), separated by a diaphragm from a low-pressure region (the driven section). Depending on the application, the driver section may be at a different temperature and filled with a different gas than the driven section. When the diaphragm ruptures, a normal shock travels into the driven section and an expansion wave travels into the driver section (12). For the current study, a constant-diameter shock tube was modeled with a driver that was allowed to vent to the atmosphere (farfield). No test article was modeled so shock tube conditions were considered unobstructed.

### Shock Tube Geometry and Baseline Mesh Description

**Figure 2:**
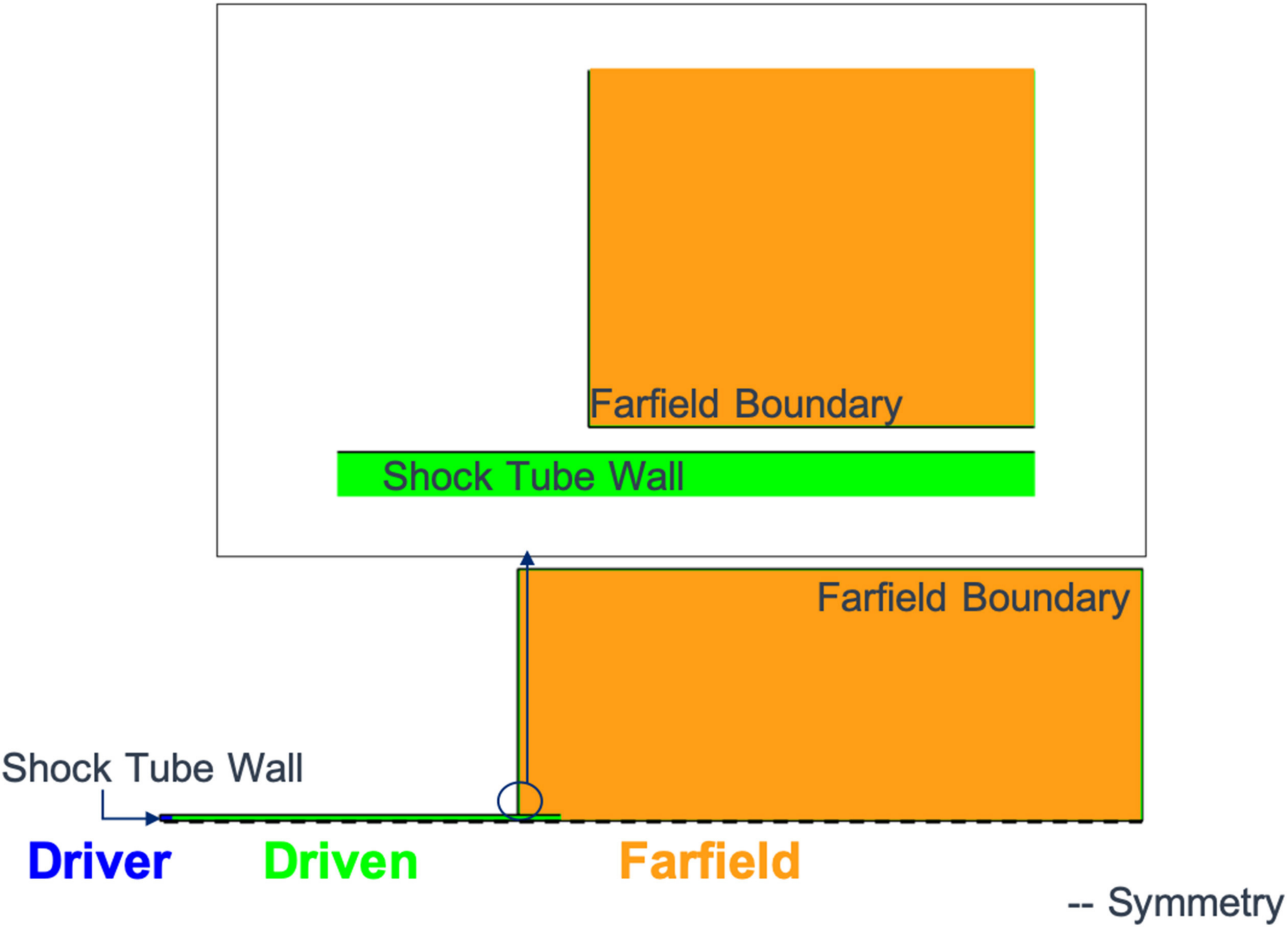
Shock tube baseline computational mesh with zoomed in view of interface between shock tube driven section and outside environment.

The shock tube geometry for these studies was a 6 inch constant-diameter shock tube with a 6 inch long driver section and a 192 inch long driven section. The computational mesh was generated in 2D using the commercial mesh generation software, Pointwise^1^. Pointwise can be used to create structured, unstructured, overset, and hybrid meshes. In this case, Pointwise was used to create a mesh with only quad elements. The mesh was created such that the bottom edge was a symmetry plane and the overall geometry was defined as 2D axisymmetric to model a circular cross section for the shock tube. Although many of the results presented in this paper could have been generated with a simpler 1D model, the 2D axisymmetric model was used to capture end-jet effects near the shock tube exit on the pressure-time history. Figure 2 shows a schematic of the 2D mesh that was used for these simulations. The zoomed-in image highlights the gap that was modeled to give the shock tube wall a finite thickness while still allowing the exiting flow to turn the corner.

### CFD Solver and Settings

CFD++^2^ (version 15.1.1.u6), a commercial CFD solver developed by Metacomp Technologies Incorporated, was used for all the simulations described in the following studies. CFD++ is a versatile and generalized code that can solve the one, two, or three-dimensional, steady or unsteady, incompressible or compressible Reynolds-Averaged Navier Stokes (RANS) equations. The code's ability to include multiple species was utilized for some of the parametric studies performed as part of this analysis. The code also has the ability to solve the Euler equations that describe inviscid flows. The code uses a finite volume formulation, a second-order total variation diminishing scheme for spatial discretization, a second-order implicit time-stepping algorithm for time discretization, and a modified Roe's Riemann solver for updating cell averages in time (13).

For the work described in this report, all simulations were set up to solve the two-dimensional (2-D) axisymmetric, unsteady, compressible fluid equations. For each time-step and grid cell, the code solves four 2-D conservation equations: mass, x-momentum, y-momentum, and energy. One or more additional equations are solved when a turbulence model is used and no additional equations are solved when the flow is modeled as inviscid.

When solving for flows with multiple species, additional N-1 equations are required, where N is the number of species. The study described in section Driver Gas Study was conducted to understand the effect of the driver gas on the pressures. At most, two species (air and helium) were modeled as one driver gas mixture, so one additional equation was solved for those simulations.

The flow through a shock tube is unsteady. The implicit (backward Euler), dual time-stepping algorithm was used to advance the solution from one physical time-step to another. At each global iteration (or time-step) the dual time-stepping algorithm iteratively solves the governing equations for a predefined number of inner iterations or until a predefined convergence criterion is satisfied. For simulating unsteady flows, the selection of the physical time-step is critical and depends on the velocities in the flow and the size of the smallest grid cell. If the time-step chosen is too large, the flow solution could become unstable and unphysical. If the time-step is too small, then the overall simulation (wall-clock) time can become very large. These issues are exacerbated when dealing with complex flows because of the need to have computational grids with many computational cells. A time-step study (results not shown here for brevity) was conducted for each candidate grid and is described later. Most simulations were conducted to achieve at least 15 ms of flow time. The maximum number of inner iterations were set to 20 to achieve a convergence of two orders of magnitude. Note that order of convergence in this context refers to the magnitude of the change between the answer at an inner-iteration relative to the magnitude of the initial guess. Early in the simulations, the code required 15–20 inner iterations to reach convergence; however, after the initial high-gradient transient flow decays, only 6–7 inner iterations were required for convergence.

**Figure 3:**
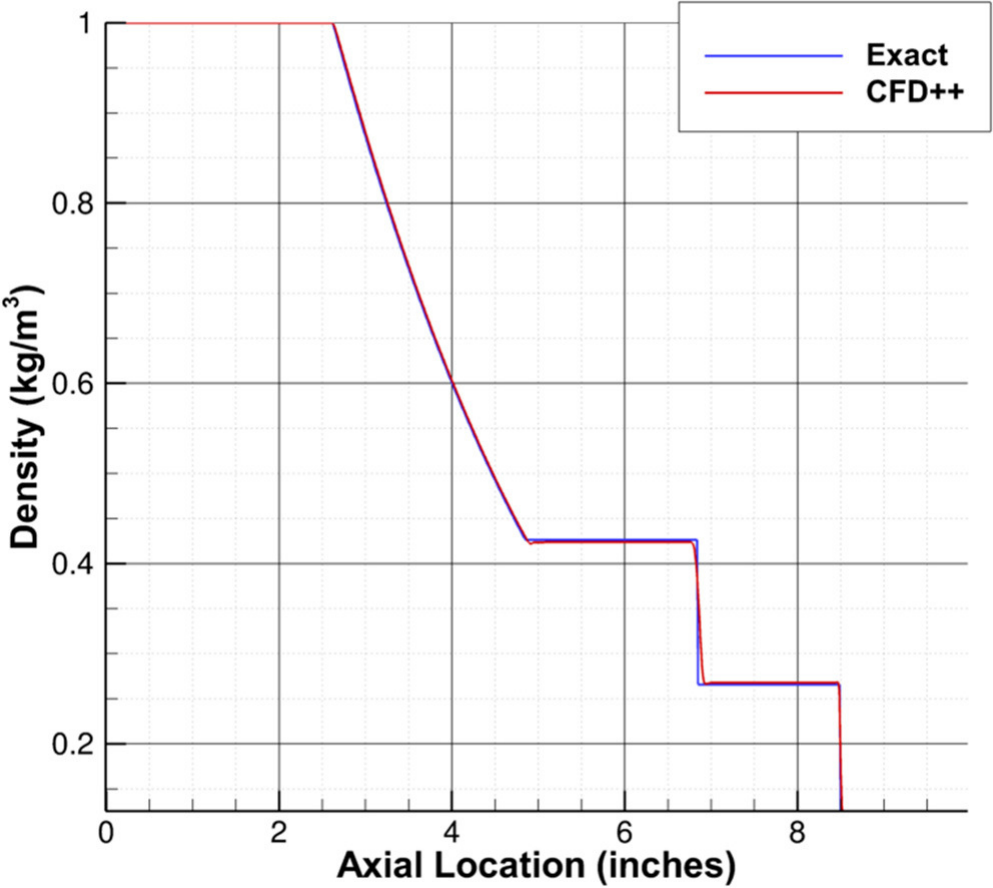
Comparison of density predicted by CFD++ and the exact solution for sod shock tube problem.

Prior to conducting simulations with the shock tube geometry and conditions of interest, the numerical schemes in CFD++ used for this work were validated against the Sod shock tube problem to ensure that the physics were being captured accurately (14). Figure 3 shows that the density predicted by CFD++ compares well with the exact solution.

### Initial and Boundary Conditions

Each CFD simulation requires initial conditions (ICs) and boundary conditions (BCs). Here, the driver section was initialized with the driver gas at a given gauge pressure (gauge burst pressure) of either 100, 150, or 200 psig. The remaining ICs are shown in Table 1.

**Table 1:** Initial conditions.

**Region**	**Absolute pressure (psi)**	**Temperature (Kelvin)**	**XYZ velocity (ft/s)**
Driver	Burst pressure	295	(0, 0, 0)
Driven	14.7	295	(0, 0, 0)
Farfield	14.7	295	(0, 0, 0)

At all solid surfaces (shock tube walls), boundaries were assumed isothermal. At the farfield boundaries, characteristic inflow/outflow conditions were applied with pressure and temperature set at 14.7 psi and 295 K, respectively. Simulations were conducted assuming a symmetry in the Z = 0 plane. A symmetry BC was used, which acts as an inviscid wall BC where only tangential flow is allowed and the normal (to the symmetry plane) component of velocity is zero. All simulations were conducted using a 6 inch long driver section, a gauge burst pressure of 150 psig, and Helium driver gas unless otherwise noted.

### Data Analysis and Post-processing

Selected locations, as a function of driver length, along the shock tube centerline were used to probe the flow. Data at these locations were taken at every time-step in order to find the pressure history at each probe location, and the maximum pressure at each location was calculated. The time at which this peak pressure occurs, t_pmax_, was also recorded. Flow-field contours of the symmetry plane were saved every 100 time-steps.

## CFD Pre-Processing: Time-Step, Mesh, and Turbulence Model Independence

Mesh and time-step resolution studies were conducted to ensure that all results were mesh and time-step independent. An under-resolved mesh would artificially dissipate the shock, and a time-step that is too large could reduce the stability of the simulations leading to spurious oscillations, particularly near discontinuities such as shocks (Gibbs phenomena) (15–17). Similarly, a mesh that was too coarse would be unable to resolve the shock, and a mesh that was too fine would increase the simulation's runtime significantly. Because the flow along the shock tube centerline was of more interest than the flow near the wall, where the effects of viscosity are more significant, the inviscid equations were solved and the mesh resolution was only varied within the tube and in the axial direction. For each candidate mesh, a time-step independence study was first conducted. A simulation was considered time-step independent when the predicted peak pressure and shock arrival time were within two percent of any simulation with a smaller time-step. Additionally, any time-steps that resulted in non-physical oscillations in the pressure-time history were disqualified from consideration. A time-step independent solution from each mesh was then compared to select a baseline mesh. This mesh resolution study found that regardless of the computational mesh used, the time of arrival for the shock and maximum peak pressures remained within two percent for each of the simulations. A mesh containing approximately 94,000 computational cells and an axial spacing of about 0.1 inches was selected as the baseline mesh with a time-step of 1 μs. After the mesh-independence and time-step independence studies were completed, a turbulence-model study was also conducted to ensure viscosity did not significantly change the results. The turbulence models did not significantly impact these results. The impact to shock arrival time was <1% and the effect on peak pressure was <5%. Therefore, the remainder of the simulations were conducted assuming an inviscid flow field to reduce the average simulation runtime. On average, the remainder of the simulations took 3 hours to run using single-node 16-core machines.

## Results

### Driver Length Study

A study was conducted to examine the effect of different driver lengths (2, 3, 6, 12, and 18 inches) on the shock evolution. Pressure traces were compared for this study at multiple probe locations based on non-dimensionalizing the probe's distance from the driver/driven interface to the driver length, hereafter referred to as λ. These pressure traces allow us to compare the shocks at a variety of locations and understand where a Friedlander profile can be achieved based on driver length.

**Figure 4:**
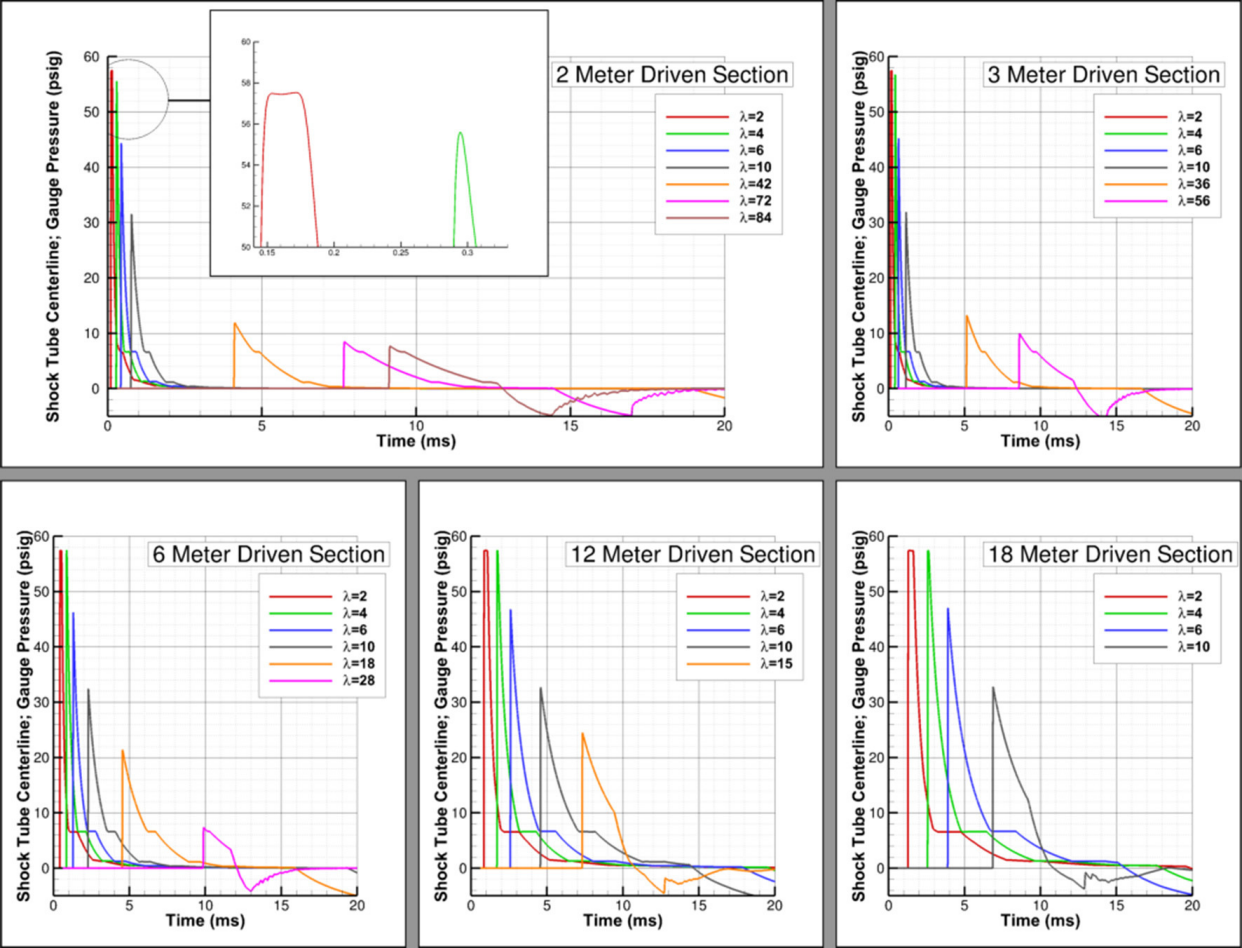
Driver length study; pressure traces shown for various probe locations for each simulation with 2, 3, 6, 12, and 18 inch driver lengths.

Figure 4 shows the formation of flat-top waves when the probe is two driver lengths from the driver/driven interface (λ = 2) regardless of driver length. A minimum λ of 4 is needed before a wave displaying an immediate exponential decay similar to the Friedlander profile is formed. However, the decay does not remain exponential for the entirety of the positive-phase duration. There is no point along the driven section where the waves are fully representative of a Friedlander wave. As the probe location moves farther downstream, the peak pressure decreases, but the peak pressure remains a function of λ. For example, Figure 4 shows that the peak pressure is approximately the same 10 driver lengths down the driven section for each simulation even though these are different dimensional distances.

**Figure 5:**
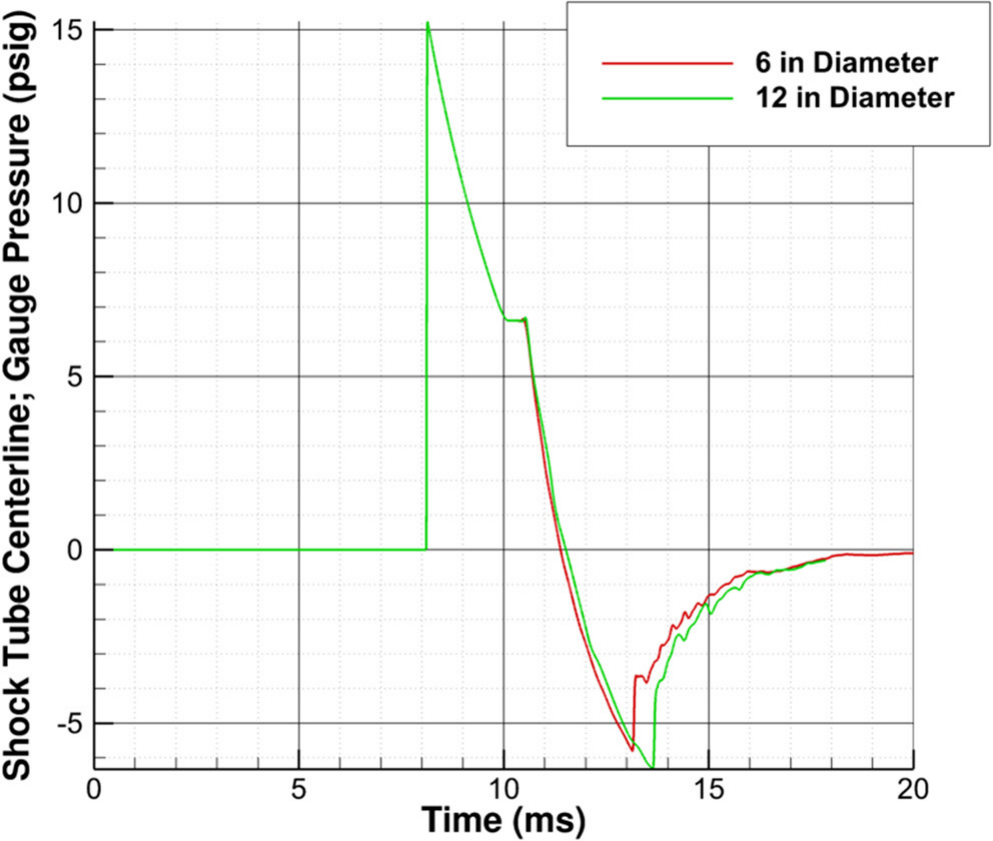
Effect of shock tube diameter (6 and 12 inches) on pressure-time history inside driven section 15 inches from shock tube exit plane.

As the waves approach the shock tube exit, the resulting pressure signature is once again less representative of a Friedlander wave. The point at which this occurs appears not to be a function of driver length, but instead a function of proximity to the shock tube exit. Yu et al. found that this critical distance was based on the shock tube diameter (9). For the simulations conducted, it appears that testing must be conducted at least two shock tube diameters away from the exit plane. Figure 5, which shows pressures 15 inches inside the driven section from the shock tube exit plane for two different shock tube diameters, appears to verify this conclusion. There are oscillations in the pressure-time history for the 12 inch diameter shock tube (15 inches is 1.25 shock tube diameters) but not the 6 inch diameter shock tube (15 inches is 2.5 shock tube diameters), and the results appear identical otherwise. This study shows that the critical position in which the test article should be placed is at least four driver lengths from the driver/driven interface and two shock tube diameters from the exit plane.

### Burst Pressure Study

In a laboratory experiment, a burst pressure study is one that tests the effect of the choice of the membrane on the pressure history. Different types of membranes burst at different pressures and, thus, the relation between the burst pressure and test-section peak pressure is observed. For our studies, it is necessary to understand the effect of membrane burst pressure on the positive phase duration of the wave. If the positive phase duration increases with the peak pressure, the overall impulse may increase significantly compared to a situation where the peak pressure increased and the positive phase duration did not.

**Figure 6:**
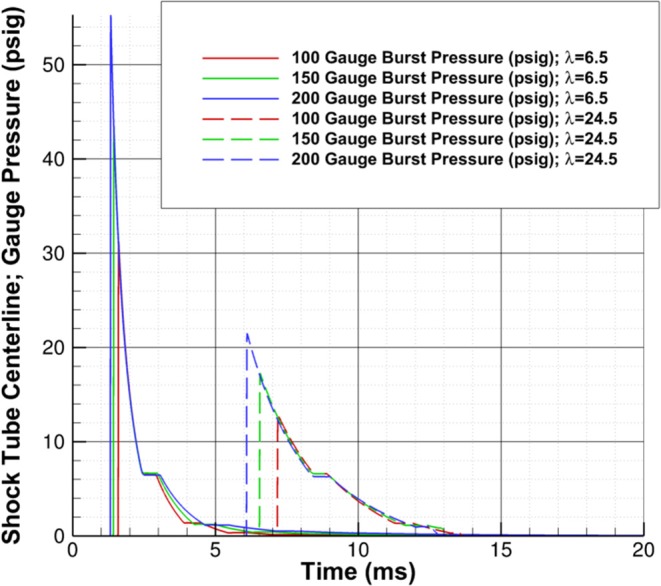
Burst pressure study; pressure-time history shown for burst pressures of 100, 150, and 200 psig at λ of 6.5 and 24.5.

The results of the burst pressure study, Figure 6, show that the peak pressure increases as the membrane burst pressure increases, but the recovery percentage, $\frac{P_{Peak}}{P_{Burst}}$, decreases as the burst pressure increases. In addition, the peak pressure decreases more rapidly as the wave travels downstream for higher burst pressures. Finally, the higher burst pressures do increase the positive phase duration of the wave, suggesting that the impulse will be greatly increased, although the decay rate of the waves are nearly identical. The recovery percentage at two different points in the driven section is in Table 2.

**Table 2:** Peak pressure recovery vs. burst pressure.

**Burst pressure (psig)**	**Peak pressure (psig); λ = 6.5**	**Pressure recovery %; λ = 6.5**	**Peak pressure (psig); λ = 24.5**	**Pressure recovery %; λ = 24.5**
100	31.2	31.2	12.8	12.8
150	43.35	28.9	17.25	11.5
200	55.2	27.6	21.2	10.6

### Driver Gas Study

The final study conducted as part of this work was to look at the effect of using air, helium, or an equal parts mixture as the driver gas. The driver gas is an important facet of the experimental setup because IEDs and explosives may release other gases, which may alter the shock properties. In a laboratory setting, it may be less desirable to use explosive nitroanimes, including RDX, due to licensing requirements and the need for specialized equipment for storage and transport (7). Because one objective of this work is to help inform future experiments, air, helium, and an equal-parts mixture, all of which are more likely to be experimentally used, were studied for this analysis.

**Figure 7:**
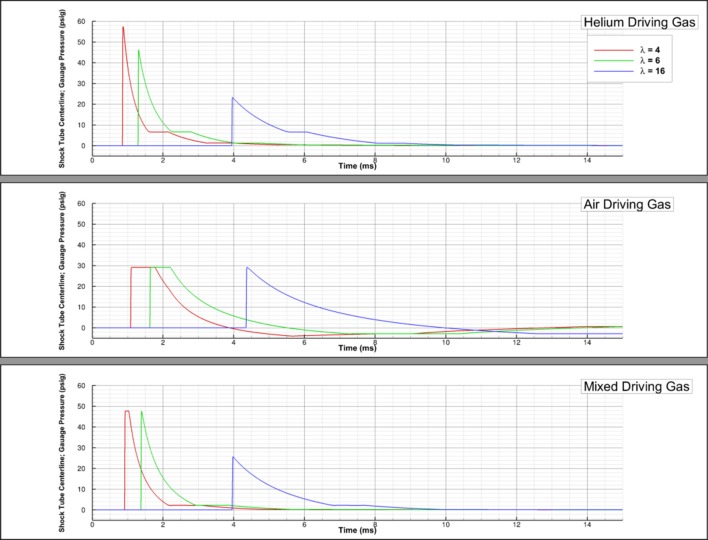
Driver gas study; pressure-time history shown for air, helium, and 50–50 air-helium mixed driving gases at λ of 4, 6, and 16.

Figure 7 shows that the shock arrival time is the earliest for a helium driver gas and the latest for an air driver gas. This is unsurprising considering that the molecular weight of helium is less than air and it also corresponds to the strength of the shock. Figure 7 also shows that, farther downstream the driven section (λ = 16), the shock arrival time for the mixed driver gas and helium driver gas is approximately the same. This can be attributed to changes in the shock strength, evidenced by the higher peak pressure predicted by the simulation with a mixed driving gas.

Figure 7 shows that a higher λ is needed for the formation of a Friedlander-like wave profile when using air or the air-helium mixture as a driving gas. Specifically, λ = 16 is needed when air is the driving gas and λ = 6 is needed for the equal parts air-helium driving gas mixture. While all the driving gases can generate the Friedlander-like wave profile, the waves generated when air is the driving gas do not have a constant pressure for any duration of the positive phase and display a negative phase duration more similar to the theoretical Friedlander wave when compared to the other driving gases.

Figure 7 also shows that the waves generated from an air driving gas may result in higher peak pressures, if the test section is sufficiently far downstream. However, the region in which the desired shock profile is generated may be small, depending on the total length of the driven section and, thus, the test section placement would need to be optimized for the conditions of the test. If helium or a mixture are used instead, researchers would likely have more freedom in test section placement.

## Discussion

The purpose of this paper was to provide design guidelines for shock tubes such that they can be used to generate blast waves. Parametric studies were conducted using CFD to characterize the primary overpressure blast environment created by a variety of laboratory shock tube designs. CFD was used to characterize changes in the flow that resulted from varying specific design features, such as: driver length, burst pressure, and driver gas. These studies demonstrated that a shock tube can be used to generate a Friedlander-like blast profile under specific conditions. The test section must be placed far enough from the driver/driven interface that there is no flat-top wave profile, and this distance depends on the driving gas. In addition, the test section must be placed some distance away from the shock tube exit such that the complex flow phenomena near the tube exit do not influence the blast profile. The chosen driving gas has a significant impact on the wave profile and can result in a wave that only looks like the Friedlander wave profile for a portion of the positive-phase duration.

Experimentalists using shock tubes to test the impact of overpressure on the brain will have to balance two major goals—to generate both a Friedlander-shaped blast profile and a sufficiently high peak pressure in the test section to correlate the results of free-field testing for a corresponding amount of explosive. Regardless of driving gas, experimentalists may be able to meet peak pressure requirements by selecting a diaphragm material that will not burst until higher pressures. Higher burst pressures did not change the rate of decay of the waves and so the peak pressure to impulse relationship should remain the same. If this option is utilized, the sublinear relationship between burst pressure and the resultant peak pressure of the wave is important to keep in mind. In the absence of being able to change the shock tube diaphragm material, a test section closer to the driver/driven interface (minimum λ = 4) and helium as the driving gas will result in the highest peak pressures. However, this will be at the cost of not matching the Friedlander profile as well and higher impulses due to the longer positive phase duration. This option could result in incorrect assumptions about how injuries occur in the field. However, for an already-built shock tube with a test section at least 16 driver lengths downstream, air as the driving gas is more likely to result in both a more representative wave and a higher peak pressure due to the higher rate of decay in peak pressure as the wave travels downstream when helium is the driving gas. An optimized air-helium mixture for the driver gas may provide both a Friedlander shock profile and required peak pressures in the test section.

A finding of the driver length study was that testing near the end of the shock tube will result in waves that are not representative of a Friedlander wave. Furthermore, the shock tube diameter, not the driver length, appears to be the key parameter related to how far the test section should be placed from the end of the shock tube. For the simulations conducted, two shock tube diameters from the exit plane is when end-jet effects began to change the wave profile. It is possible that asymmetries in the 3D environment outside of the shock tube such as the ground or floor may also impact end-jet effects and could change the conclusions of this study. CFD could be used to check for end-jet effects and analysts should look for oscillations during the decay of the wave.

In conclusion, the studies demonstrated that shock tubes are a viable method of generating Friedlander-shaped blast profiles. However, careful attention must be paid to the shock tube and experimental design to ensure this profile is achieved while meeting minimum peak pressure requirements in a test section. Experimentalists will need to balance placing the test section sufficiently far from the driver/driven interface such that a Friedlander-like wave profile is formed, which will depend on the driving gas, while placing the test section far enough from the exit of the shock tube such that end-jet effects do not influence the wave. CFD can be used to help design the shock tube and provide pre-test predictions to verify that all requirements will be met.

## Data Availability Statement

The datasets generated for this study are available on request to the corresponding author.

## Author Contributions

RK conducted the analysis, analyzed the results, and wrote the manuscript. AN provided technical guidance and developed the process for conducting the analysis.

### Conflict of Interest

The authors declare that the research was conducted in the absence of any commercial or financial relationships that could be construed as a potential conflict of interest.

## Footnotes

^1^The software Pointwise and more information about it is available at www.pointwise.com.

^2^The software CFD++ and more information about it and about Metacomp Technologies Incorporated can be found at www.metacomptech.com.
